# Risk factors for intracerebral hemorrhage in small-vessel disease and non-small-vessel disease etiologies—an observational proof-of-concept study

**DOI:** 10.3389/fneur.2024.1322442

**Published:** 2024-03-07

**Authors:** Philipp Arndt, Christian Chahem, Michael Luchtmann, Jan-Niklas Kuschel, Daniel Behme, Malte Pfister, Jens Neumann, Michael Görtler, Marc Dörner, Marc Pawlitzki, Robin Jansen, Sven G. Meuth, Stefan Vielhaber, Solveig Henneicke, Stefanie Schreiber

**Affiliations:** ^1^Department of Neurology, Otto-von-Guericke University, Magdeburg, Germany; ^2^German Center for Neurodegenerative Diseases (DZNE) within the Helmholtz Association, Magdeburg, Germany; ^3^Department of Neurosurgery, Paracelsus-Klinik, Zwickau, Germany; ^4^Department of Neurosurgery, Otto-von-Guericke University, Magdeburg, Germany; ^5^Department of Neuroradiology, Otto-von-Guericke University, Magdeburg, Germany; ^6^Department of Consultation-Liaison-Psychiatry and Psychosomatic Medicine, University Hospital Zurich, University of Zurich, Zurich, Switzerland; ^7^Department of Neurology, Heinrich-Heine-University, Düsseldorf, Germany; ^8^Center for Behavioral Brain Sciences (CBBS), Magdeburg, Germany

**Keywords:** intracerebral hemorrhage, cerebral small-vessel disease, cerebral amyloid angiopathy, hypertensive arteriopathy, hypertension, trauma, vascular malformation, brain tumor

## Abstract

**Background:**

Sporadic cerebral small-vessel disease (CSVD), i.e., hypertensive arteriopathy (HA) and cerebral amyloid angiopathy (CAA), is the main cause of spontaneous intracerebral hemorrhage (ICH). Nevertheless, a substantial portion of ICH cases arises from non-CSVD etiologies, such as trauma, vascular malformations, and brain tumors. While studies compared HA- and CAA-related ICH, non-CSVD etiologies were excluded from these comparisons and are consequently underexamined with regard to additional factors contributing to increased bleeding risk beyond their main pathology.

**Methods:**

As a proof of concept, we conducted a retrospective observational study in 922 patients to compare HA, CAA, and non-CSVD-related ICH with regard to factors that are known to contribute to spontaneous ICH onset. Medical records (available for *n* = 861) were screened for demographics, antithrombotic medication, and vascular risk profile, and CSVD pathology was rated on magnetic resonance imaging (MRI) in a subgroup of 185 patients. The severity of CSVD was assessed with a sum score ranging from 0 to 6, where a score of ≥2 was defined as advanced pathology.

**Results:**

In 922 patients with ICH (median age of 71 years), HA and CAA caused the majority of cases (*n* = 670, 73%); non-CSVD etiologies made up the remaining quarter (*n* = 252, 27%). Individuals with HA- and CAA-related ICH exhibited a higher prevalence of predisposing factors than those with non-CSVD etiologies. This includes advanced age (median age: 71 vs. 75 vs. 63 years, *p* < 0.001), antithrombotic medication usage (33 vs. 37 vs. 19%, *p* < 0.001), prevalence of vascular risk factors (70 vs. 67 vs. 50%, *p* < 0.001), and advanced CSVD pathology on MRI (80 vs. 89 vs. 51%, *p* > 0.001). However, in particular, half of non-CSVD ICH patients were either aged over 60 years, presented with vascular risk factors, or had advanced CSVD on MRI.

**Conclusion:**

Risk factors for spontaneous ICH are less common in non-CSVD ICH etiologies than in HA- and CAA-related ICH, but are still frequent. Future studies should incorporate these factors, in addition to the main pathology, to stratify an individual’s risk of bleeding.

## 1 Introduction

Intracerebral hemorrhage (ICH) is a severe form of acute stroke, with significant public health implications due to high mortality and disability rates, accounting for nearly half of all stroke-related morbidity and deaths (1, 2). Spontaneous ICH arises primarily from cerebral small-vessel disease (CSVD), particularly hypertensive arteriopathy (HA) and cerebral amyloid angiopathy (CAA). These conditions result in chronic microvascular dysfunction. Hemorrhage can also result from traumatic brain injury, vascular malformations, or brain tumors, collectively referred to as non-CSVD ICH etiologies. Some studies have compared HA- and CAA-associated ICH, claiming that patients with CAA are older, and present more frequently with cortical superficial siderosis, strictly lobar cerebral microbleeds (CMBs), and severe centrum semiovale perivascular spaces (PVS), whereas patients with HA, reveal more often arterial hypertension, severe basal ganglia PVS and strictly deep or mixed deep and lobar CMBs (2, 3). Non-CSVD etiologies have often been excluded from these comparisons, and consequently, they remain underexamined in terms of vascular risk factors, antithrombotic medication usage, and the presence and severity of CSVD as potential contributors to all-cause ICH. In this study, we investigated 922 patients with first-ever ICH, aiming to determine the prevalence of factors associated with increased bleeding risk, including vascular risk profile, antithrombotic medication use, and CSVD severity in HA-, CAA-, and non-CSVD-associated ICH. We hypothesize that these factors, either individually or in combination, may contribute to a hemorrhage-prone cerebral microvasculature, thus playing a role in the onset of non-CSVD ICH.

## 2 Methods

### 2.1 Study design and participants

This retrospective observational study utilized the patient database of the neurology and neurosurgery departments at Otto-von-Guericke University, Magdeburg. Between March 2003 and October 2018, 922 adult patients with first-ever ICH and computed tomography (CT) scans within 24 h of symptom onset were identified. ICH location was categorized based on the Cerebral Hemorrhage Anatomical Rating Instrument, including lobar, deep (basal ganglia, thalamus, brainstem, and mixed deep/lobar), cerebellar, and adult primary intraventricular hemorrhage (referred to as ventricular ICH with blood confined to the ventricular system without associated parenchymal hemorrhage).

### 2.2 ICH etiology

Intracerebral hemorrhage was caused by HA, CAA, or non-CSVD etiologies, including trauma, vascular malformations, and brain tumors. All diagnoses were made by experienced neuroradiologists (minimum 5 years of practice). In short, CSVD-related ICH was considered in case of the absence of trauma, vascular malformations, and brain tumors and was classified as caused by either CAA or HA. CAA diagnosis was based on the modified Boston criteria (4) and HA diagnosis on the existence of deep or mixed, i.e., deep and lobar, hemorrhage(s) (5). Traumatic ICH was diagnosed in case of fall, head trauma, skull fracture, and observable epidural or subdural hematoma (6). Vascular malformations comprising aneurysms, arteriovenous malformations, dural fistula, and cavernoma were detected through vascular and magnetic resonance imaging (MRI). Diagnosis of brain tumor was based on typical CT- and/or MRI features (e.g., intracranial mass lesion, breakdown of blood–brain barrier, contrast enhancement, and peritumoral edema) and histopathology. ICH cases with no clear cause and hemorrhagic transformation of ischemic stroke were excluded from this study.

### 2.3 Clinical data

Age and sex were available for all patients. Medical records were retrospectively screened for vascular risk factors and intake of antithrombotic medication at the time of ICH by CC; information was available for the majority of patients (*n* = 861, 93%; *n* = 427 HA, *n* = 204 CAA, and *n* = 230 non-CSVD). Neurovascular risk factors were defined by prior diagnosis and antihypertensive, antidiabetic, or lipid-lowering medication. Intake of two or multiple antihypertensive drugs is recommended in more severe hypertension stages (7) and therefore used as an indicator of greater hypertension severity or longer hypertensive disease duration. Additionally, we considered clinical laboratory blood tests for dyslipidemia (total cholesterol > 5.2 mmol/L, low-density lipoprotein cholesterol > 2.6 mmol/L, high-density lipoprotein cholesterol < 1.0 mmol/L, or triglycerides > 1.7 mmol/L) and type 2 diabetes (HbA1c ≥ 5.7% or fasting plasma glucose level ≥ 5.6 mmol/L). To consider a broader vascular risk, prediabetic patients were included in the type 2 diabetes group, as cerebral microvascular dysfunction and pathology are already apparent in prediabetic adults (8). Patients took either anticoagulants (warfarin, rivaroxaban, apixaban, and edoxaban), antiplatelets (aspirin, clopidogrel, and prasugrel), a combination of both, or none. Smoking was not considered as this information was available in 318 patients (34%) only. To evaluate the overall vascular risk profile of each patient, we calculated a vascular risk sum score. Each risk factor (hypertension, dyslipidemia, and type 2 diabetes), if present, was assigned one point, which was then divided by the total number of risk factors, resulting in values from 0 to 1.

### 2.4 Vascular imaging

Vascular imaging was performed in 729 (79%) patients, using CT angiography (*n* = 545, 75%), magnetic resonance angiography (*n* = 75, 10%), or digital subtraction angiography (*n* = 109, 15%). Among them, 109 (15%) were diagnosed with vascular malformations [52 aneurysms, 40 arteriovenous malformations, 13 dural fistulas, and four cavernoma (confirmed by MRI)]. The remaining 620 patients (85%) did not exhibit such vascular abnormalities. Of the 193 patients without vascular imaging, 50 (26%) had traumatic ICH, 9 (5%) had brain tumors, and 134 (69%) were suspected to have CSVD (Supplementary Table 1).

### 2.5 MRI acquisition and analysis

In total, 223 (24%) patients underwent MRI. Thirty-eight scans (*n* = 23 with CSVD-related ICH; *n* = 15 with non-CSVD-related ICH) were excluded from the analysis due to motion artifacts (*n* = 30) or missing sequences needed to complete the MRI analysis (*n* = 8), leaving *n* = 185 patients (20%, *n* = 71 HA, *n* = 37 CAA, and *n* = 77 non-CSVD) with available MRI for analysis. MRI was performed using 1.5 T (Siemens Healthineers, Erlangen, Germany; *n* = 138) or 3 T scanner (MAGNETOM Skyra, Siemens, Erlangen, Germany; *n* = 47), including the following sequences: T2*-GRE [slice thickness (ST): 3–6 mm, repetition time (TR): 493–1,421 ms, echo time (TE): 14–26 ms], FLAIR (ST: 3–6 mm, TR: 5,000–11,000 ms, TE: 77–145 ms), and T2-weighted (ST: 3–6 mm, TR: 1,040–11,121 ms, TE: 74–128 ms). MRI analysis of all patients was performed by one trained investigator (CC), blinded to clinical information, using Mango software for DICOM images (Supplementary Table 2).

### 2.6 CSVD markers

Magnetic resonance imaging markers of CSVD severity were rated according to Standards for Reporting Vascular Changes on Neuroimaging (9, 10). CMBs were defined as small (diameter 2–5 mm, maximum up to 10 mm) round or oval hypointense lesions on T2*-GRE, not visible on T2-weighted sequences. They were categorized by applying the Microbleed Anatomical Rating Scale (11). Curvilinear residues of chronic blood products following the cortical surface distinct from vessels visible as linear hypointensities on T2*-GRE sequences were defined as cortical superficial siderosis. Cortical superficial siderosis was classified as not existent, focal (1 non-adjacent sulci with cortical superficial siderosis in each hemisphere or < 3 immediately adjacent sulci with cortical superficial siderosis), or multifocal (2 non-adjacent sulci with cortical superficial siderosis in each hemisphere or > 3 immediately adjacent sulci with cortical superficial siderosis). FLAIR sequences were applied to rate the severity of white matter hyperintensities (WMH) in deep and periventricular regions according to the Fazekas scale (12) and to count the number of lacunes (defined as small, ovoid, hyperintense cavities between 3 and 15 mm in diameter) (13) in deep and lobar regions. PVSs were rated on T2-weighted sequences in the basal ganglia and centrum semiovale using a validated four-point visual rating scale (0 = none, 1 = <10, 2 = 10–20, 3 = 21–40, and 4 = >40) (14). In 15 randomly chosen MRIs, all CSVD markers were scored twice (by CC and MP) with excellent intra- and inter-rater reliability of the global CSVD severity (intraclass correlation coefficients: 0.92 and 0.9, respectively). Both raters were blinded against clinical data.

### 2.7 CSVD severity

We used established ordinal scales to evaluate the global CSVD, HA, and CAA burden on MRI (14). The global CSVD severity was scored from 0 to 6, with one point being allocated for the presence of (i) lacunes, (ii) 1–4 CMB, (iii) severe basal ganglia PVS (>20), and (iv) moderate WMH (sum of deep + periventricular WMH grade 3–4). Two points were allocated for the presence of (i) >5 CMB or (ii) severe WMH (sum of deep + periventricular WMH grade 5–6). For HA severity, we scored from 0 to 4, with one point being allocated for the presence of (i) lacunes, (ii) deep WMH grade 2–3 or periventricular WMH grade 3, (iii) the presence of ≥1 deep CMB, and (iv) moderate-to-severe basal ganglia PVS (> 10). CAA severity was scored from 0 to 6, with one point each for the presence of (i) 1–4 lobar CMB, (ii) severe centrum semiovale PVS (>20), (iii) deep WMH ≥ 2 or periventricular WMH grade 3, and (d) focal cortical superficial siderosis. We allocated two points for (i) ≥5 lobar CMB and (ii) multifocal cortical superficial siderosis.

### 2.8 Standard protocol approvals, registrations, and patient contents

The local ethics committee of the medical faculty, Otto-von-Guericke University, Magdeburg (No. 28/16, addendum 07/2018) approved this retrospective study. Informed consent was obtained from all patients for anonymized retrospective analysis of their data conducted for clinical diagnostics.

### 2.9 Statistical analysis

The Shapiro–Wilk test was used to assess the Gaussian distribution of the data. Demographics, clinical, and MRI variables were compared between different ICH locations and/or etiologies in univariate analyses. Continuous and ordinal variables were expressed as median (interquartile range, IQR) and compared using the Kruskal–Wallis test. Categorical variables were expressed as numbers (percentages) and compared using the chi-square test. Both tests were corrected for multiple comparisons using the Bonferroni *post-hoc* testing. Multivariable linear regression was performed to test independent associations with CSVD severity. Stepwise forward variable selection (*p* < 0.05) was used to generate a minimally adjusted model. An adjusted *p* value of <0.05 was considered significant. Analyses were performed using IBM SPSS Statistics 24.0 software.

## 3 Results

**Figure 1:**
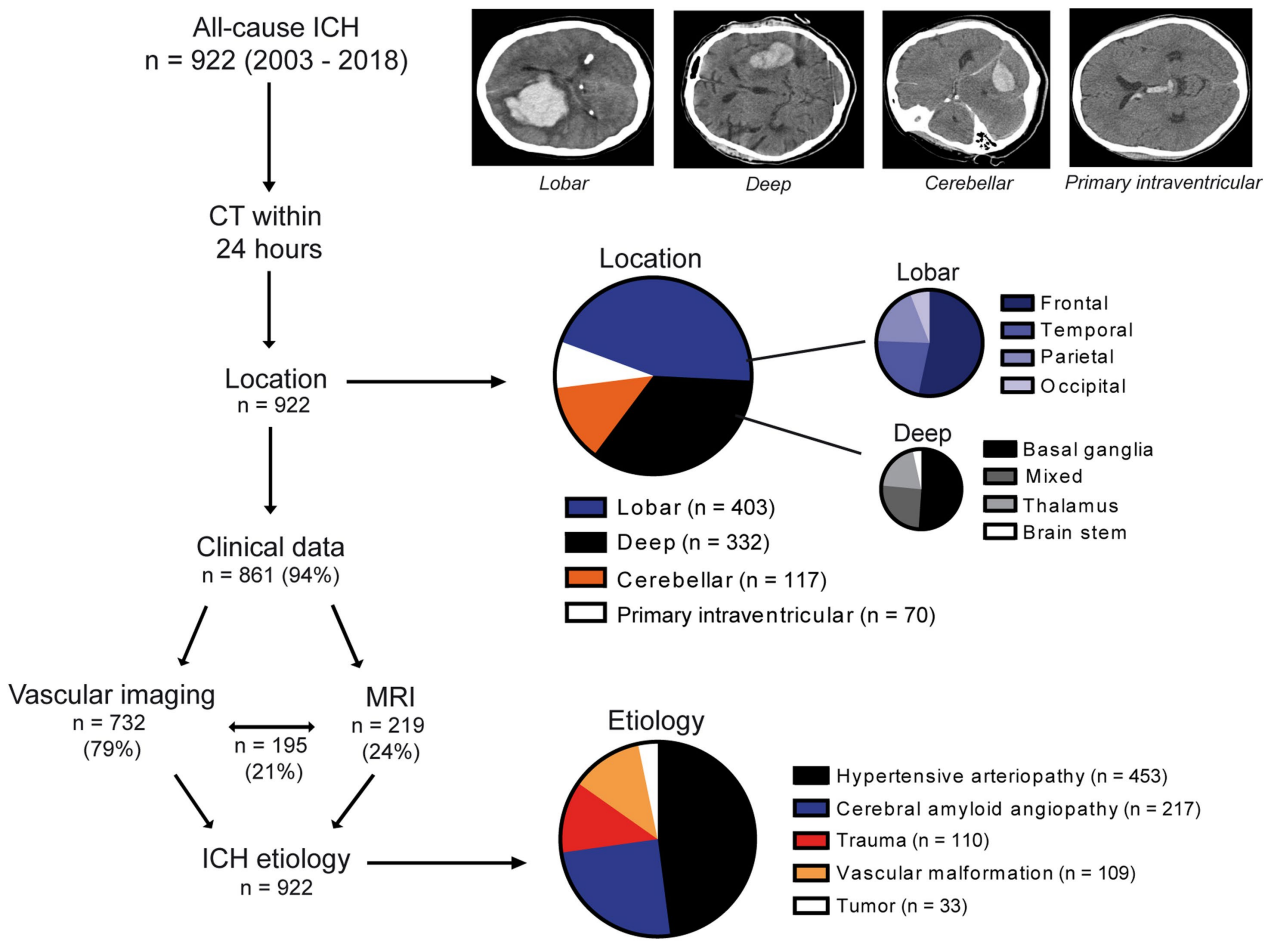
Study cohort, diagnostic workflow, and data overview. In this retrospective study, 922 patients with first-ever ICH were included. The diagnostic workflow comprised an initial CT scan (*n* = 922), clinical (*n* = 861), and imaging data (vascular imaging, *n* = 732; MRI, *n* = 219; and both, *n* = 195). ICH location was categorized as lobar, deep, cerebellar, or primary intraventricular. Lobar ICH was further subcategorized as frontal, temporal, parietal, or occipital and deep ICH as affecting primarily the basal ganglia, thalamus, brain stem, or several brain regions (i.e., lobar and deep), namely, mixed ICH. After the assessment of all diagnostic data, generated in the workflow, ICH etiology was determined in all cases (*n* = 922) as caused by either hypertensive arteriopathy, cerebral amyloid angiopathy, trauma, vascular malformation, or tumor. CT, Computed tomography; ICH, Intracerebral hemorrhage; MRI, Magnetic resonance imaging.

We enrolled 922 patients with first-ever ICH, with a median age of 71 years (IQR 60–78); 522 (57%) were men. Patient characteristics and workflow are shown in Figure 1 and Table 1. CSVD was the predominant ICH etiology (*n* = 670, 73%), with 453 (49%) attributed to HA and 217 (24%) to CAA. Of the latter, 141 (65%) were possible CAA, 49 (23%) probable CAA, and 27 (12%) probable CAA with supporting histopathology. HA was the major cause for deep (*n* = 309, 93%), cerebellar (*n* = 94, 80%), and ventricular ICH (*n* = 50, 71%), while CAA was the most frequent cause for lobar ICH (*n* = 217, 54%). Non-CSVD etiologies included trauma, vascular malformations, and brain tumors, which caused the remaining third of ICH cases (*n* = 252, 27%), mainly accounting for the remainder of lobar ICH (*n* = 186, 46%). A comparison of ICH location is displayed in Supplementary Table 3.

**Table 1:** Demographics and clinical data in different ICH etiology subgroups.

	Total	HA	CAA	Non-CSVD	*Group analysis*	*Subgroup comparisons*
Demographics	*n = 922*	*n = 453*	*n = 217*	*n = 252*	*p* value	*p* value
Age	71 (60–78)	71 (61–77)	75 (68–80)	63 (51–74)	***p* < 0.0001**	***CAA* vs. *HA, non-CSVD: adj. p < 0.0001***
						***HA* vs. *non-CSVD: adj. p < 0.0001***
Male sex	522 (57%)	260 (57%)	112 (52%)	150 (60%)	*p* = 0.20	
Vascular risk factors	*n = 861*	*n = 427*	*n = 204*	*n = 230*		
Vascular risk sum score	0.33 (0.00–0.67)	0.33 (0.00–0.67)	0.33 (0.00–0.67)	0.33 (0.00–0.33)	***p* < 0.0001**	***HA, CAA* vs. *non-CSVD: adj. p < 0.001***
Three vascular risk factors	71 (8%)	50 (12%)	12 (6%)	9 (4%)	***p* = 0.006**	***HA: adj. p = 0.001***
Hypertension	450 (52%)	240 (56%)	118 (58%)	92 (40%)	***p* < 0.0001**	***Non-CSVD: adj. p = 0.0001***
≥ 2 anti-hypertensive drugs	300 (35%)	180 (42%)	63 (31%)	57 (25%)	***p* < 0.0001**	***HA: adj. p < 0.0001***
						***Non-CSVD: adj. p = 0.001***
Dyslipidemia	253 (29%)	147 (34%)	64 (31%)	42 (18%)	***p* < 0.0001**	***Non-CSVD: adj. p < 0.0001***
						***HA: adj. p = 0.008***
Intake of statins^a^	150 (59%)	76 (52%)	49 (77%)	25 (59%)	***p* = 0.003**	***CAA: adj. p = 0.006***
						***HA: adj. p = 0.02***
Type 2 diabetes	197 (23%)	121 (28%)	36 (18%)	40 (17%)	***p* = 0.001**	***HA: adj. p = 0.0009***
Antithrombotics	*n = 862*	*n = 427*	*n = 204*	*n = 230*		
Antithrombotics	258 (30%)	140 (33%)	75 (37%)	43 (19%)	***p* < 0.0001**	***Non-CSVD: adj. p < 0.0001***
Anticoagulants	149 (17%)	83 (19%)	41 (20%)	25 (11%)	***p* = 0.01**	***Non-CSVD: adj. p = 0.008***
Antiplatelets	97 (11%)	51 (12%)	30 (15%)	16 (7%)	***p* = 0.03**	
Anticoagulants and Antiplatelets	12 (1%)	6 (1%)	4 (2%)	2 (1%)	*p* = 0.64	

Data are presented as median (interquartile range) or number (%). The percentage is given in relation to the specific subgroup. Group comparisons between HA-, CAA-, and non-CSVD-related ICH were conducted by applying the Kruskal–Wallis test for continuous/ordinal variables and the chi-square test for categorical variables. *p*-values from pairwise *post-hoc* tests were Bonferroni adjusted for multiple comparisons. For categorical variables, the *post-hoc* test, Bonferroni adjusted for multiple comparisons, was used to identify which particular subgroups were significantly different from the entire cohort. A (adjusted) *p*-value of ≤ 0.05 was considered significant and highlighted in bold. ^a^Percentage is given in relation to the number of dyslipidemia patients. CAA, Cerebral amyloid angiopathy; CSVD, Cerebral small-vessel disease; HA, Hypertensive arteriopathy; and ICH, Intracerebral hemorrhage.

Cerebral amyloid angiopathy-related ICH patients were the oldest (median age 75 years), while non-CSVD ICH cases had the youngest age (median age 63 years) (CAA vs. HA, non-CSVD, and HA vs. non-CSVD: adj. *p* < 0.0001). The vascular risk profile was most severe in HA-related ICH, evident by high rates of three risk factors (HA: adj. *p* = 0.001), intake of multiple antihypertensive drugs (HA: *p* < 0.0001), and type 2 diabetes (HA: *p* = 0.0009). It was lowest in non-CSVD ICH patients due to a lower rate of hypertension (non-CSVD: adj. *p* = 0.0001), intake of multiple antihypertensive drugs (non-CSVD: adj. *p* = 0.001), and dyslipidemia (non-CSVD: adj. *p* = 0.0001). The intake rate of antithrombotic medication was lower in non-CSVD ICH than in HA and CAA (non-CSVD: adj. *p* < 0.0001) (Table 1).

We compared global CSVD, HA, and CAA severity on MRI among the three ICH etiologies in 185 (20%) patients. HA- and CAA-related ICH patients had higher global CSVD severity scores driven by a higher load of CMB, WMH, and PVS (for each: HA, CAA vs. non-CSVD: adj. *p* < 0.0001). Markers that are specific for CAA, e.g., strictly lobar CMB, high degree of centrum semiovale PVS, or cortical superficial siderosis, were common in CAA-related ICH but comparably rare in HA- and non-CSVD ICH (for both CAA: adj. *p* < 0.0001). HA-specific markers, e.g., lacunes, a high degree of basal ganglia PVS, or deep CMB, occurred most frequently in HA-related ICH. Interestingly, a high degree of basal ganglia PVS and lacunes were frequently found in CAA-related ICH patients as well, suggesting that CAA pathology might be specific to CAA-related ICH, whereas HA pathology might also exist concomitantly to CAA pathology.

In particular, two-thirds of non-CSVD ICH patients had at least one CMB (*n* = 46, 60%), and more than one-third had mixed deep and lobar CMB (*n* = 29, 38%), resulting in an overall high load of CMB with a median of 4 (IQR 0–10). The presence of deep CMBs in 42 (55%) and lacunes in 6 (8%) non-CSVD ICH patients also points to an accompanying HA pathology in a substantial portion of non-CSVD ICH cases, whereas CAA-specific MRI markers were rather absent (Table 2). CSVD severity, as well as single neuroimaging features were similar among trauma-, vascular malformation- and brain tumor related ICH, suggesting that the etiology in non-CSVD ICH has no effect on concurrent CSVD burden (Supplementary Table 4). In multivariable linear regression, all CSVD scores were associated with increasing age, and hypertension was independently associated with increasing global CSVD (*B* = 0.52 [95% CI 0.03–1.00], *p* = 0.04) and HA severity (*B* = 0.37 [95% CI 0.04–0.70], *p* = 0.03), which was driven by higher numbers of lacunes and basal ganglia PVS (Table 3; Supplementary Table 5).

**Table 2:** Quantification of CSVD severity according to STRIVE in ICH due to CSVD and non-CSVD etiology.

	Total	HA	CAA	Non-CSVD	*Group analysis*	*Subgroup comparison*
MRI scores	*n = 185*	*n = 71*	*n = 37*	*n = 77*	*p* value	*p* value
Global CSVD score	2 (1–4)	3 (2–5)	3 (2–4)	2 (0–2)	***p < 0.0001***	***HA, CAA* vs. *Non-CSVD: adj. p < 0.0001***
Cerebral microbleeds						
Number of CMB	9 (2–17)	12 (5–19)	14 (10–25)	4 (0–10)	***p < 0.0001***	***CAA, HA* vs. *Non-CSVD: adj. p < 0.0001***
Strictly lobar CMB	41 (22%)	0 (0%)	37 (100%)	4 (5%)	***p < 0.0001***	***CAA: adj. p < 0.0001***
Strictly deep CMB	36 (19%)	23 (32%)	0 (0%)	13 (17%)	***p = 0.0004***	***HA: adj. p = 0.006***
						***CAA: p = 0.006***
Mixed CMB	66 (36%)	37 (52%)	0 (0%)	29 (38%)	***p < 0.0001***	***HA: adj. p = 0.001***
						***CAA: p < 0.0001***
White matter hyperintensities						
Periventricular WMH score	1 (1–2)	2 (1–3)	2 (1–3)	1 (0–1)	***p < 0.0001***	***HA, CAA* vs. *Non-CSVD: adj. p < 0.0001***
Deep WMH score	1 (0–2)	1 (0–2)	1 (1–2)	0 (0–1)	***p < 0.0001***	***HA, CAA* vs. *Non-CSVD: adj. p < 0.0001***
Perivascular spaces						
Basal ganglia PVS score	1 (1–2)	2 (1–3)	2 (1–2)	1 (1–1)	***p < 0.0001***	***HA, CAA* vs. *Non-CSVD: adj. p < 0.0001***
Severe basal ganglia PVS (>20)	28 (15%)	21 (30%)	7 (19%)	0 (0%)	***p < 0.0001***	***HA: adj. p = 0.0001***
						***Non-CSVD: p < 0.0001***
Centrum semiovale PVS score	2 (1–2)	2 (1–2)	3 (2–3)	1 (1–2)	***p < 0.0001***	***CAA* vs. *HA, Non-CSVD: adj. p < 0.004***
						***HA* vs. *Non-CSVD: adj. p = 0.001***
Severe centrum semiovale PVS (>20)	34 (18%)	11 (16%)	19 (51%)	4 (5%)	***p < 0.0001***	***CAA: adj. p < 0.0001***
						***Non-CSVD: p = 0.0006***
Lacunes						
Lacune presence	30 (16%)	19 (27%)	5 (14%)	6 (8%)	***p = 0.007***	***HA: p = 0.01***
Lobar lacune presence	16 (9%)	9 (13%)	2 (5%)	5 (6%)	*p = 0.30*	
Deep lacune presence	21 (11%)	15 (21%)	5 (14%)	1 (1%)	***p = 0.01***	***non-CSVD: p = 0.008***
Cortical superficial siderosis						
Presence of cortical superficial siderosis	16 (9%)	2 (3%)	13 (35%)	1 (1%)	***p < 0.0001***	***CAA: adj. p < 0.0001***

Data are presented as median (IQR) or number (%). The percentage is given in relation to the specific subgroup. Group comparisons between HA-, CAA-, and non-CSVD-related ICH were conducted by applying the Kruskal–Wallis test for continuous/ordinal variables and the chi-square test for categorical variables. *p* values from pairwise *post-hoc* tests were Bonferroni adjusted for multiple comparisons. For categorical variables, the *post-hoc* test was used to identify which particular subgroups were significantly different from the entire cohort. A (adjusted) *p*-value of ≤0.05 was considered significant and highlighted in bold. CAA, Cerebral amyloid angiopathy; CMB, Cerebral microbleeds; CSVD, Cerebral small-vessel disease; PVS, Perivascular spaces; HA, Hypertensive arteriopathy; ICH, Intracerebral hemorrhage; IQR, Interquartile range; MRI, Magnetic resonance imaging; n, Number; WMH, White matter hyperintensities.

**Table 3:** Multivariable linear regression analysis identifies predictors of CSVD severity in ICH.

	Global CSVD score			HA-CSVD score			CAA-CSVD score		
	*B (95% CI)*	*β*	*p* value	*B (95% CI)*	*β*	*p* value	*B (95% CI)*	*β*	*p* value
Vascular risk									
Hypertension	0.52 (0.03, 1.00)	0.15	**0.04**	0.37 (0.04, 0.70)	0.15	**0.03**	0.03 (−050, 0.55)	0.01	0.73
Dyslipidemia	0.25 (−0.40, 0.90)	0.07	0.33	0.05 (−0.40, 0.50)	0.02	0.81	0.09 (−0.46, 0.64)	0.03	0.75
Intake of statins	−0.15 (−0.95, 0.65)	−0.03	0.79	−0.14 (−0.70, 0.41)	−0.04	0.79	−0.46 (−1.13, 0.21)	−0.10	0.18
Type 2 diabetes	0.33 (−0.24, 0.89)	0.09	0.17	0.27 (−0.12, 0.66)	0.09	0.16	0.34 (−0.14, 0.82)	0.09	0.16
Covariates									
HA-ICH	1.27 (0.76, 1.79)	0.35	**< 0.001**	1.02 (0.71, 1.33)	0.41	**< 0.001**	0.67 (0.24, 1.09)	0.20	**0.002**
CAA-ICH	1.39 (0.75, 2.00)	0.32	**< 0.001**	−0.02 (−0.49, 0.43)	−0.01	0.89	2.53 (2.00, 3.06)	0.63	**< 0.001**
Age	0.03 (0.01, 0.04)	0.24	**< 0.001**	0.02 (0.01, 0.03)	0.28	**< 0.001**	0.02 (0.01, 0.04)	0.20	**0.001**
Male sex	0.05 (−0.40, 0.50)	0.02	0.74	0.04 (−0.27, 0.35)	0.02	0.71	−0.04 (−0.42, 0.33)	−0.01	0.84
Adjusted *R*^2^	**0.32**			**0.33**			**0.43**		

Multivariable linear regression analyses were performed to test for independent associations with different MRI-based CSVD severity patterns, including ICH etiology, demographics, and vascular risk. Stepwise forward variable selection (*p* < 0.05) was used to generate a minimally adjusted model. Data represent regression coefficient (*B* [95% confidence interval]), standardized effects estimates (β), and the corresponding *p*-value. Associations achieving statistical significance (*p* < 0.05) are highlighted in bold. CAA, Cerebral amyloid angiopathy; CI, Confidence interval; CSVD, Cerebral small-vessel disease; HA, Hypertensive arteriopathy; and ICH, Intracerebral hemorrhage.

## 4 Discussion

This retrospective single-center observational study included 922 patients with first-ever ICH and compared, to the best of our knowledge, for the first-time, clinical characteristics and—in a subset—MRI-based CSVD severity among HA-, CAA-, and non-CSVD-related ICH. Our study emphasizes the significance of CSVD-related ICH. Compared to non-CSVD ICH, both CSVD subgroups were older and exhibited advanced CSVD, frequent systemic vascular risk factors, and antithrombotic medication, overall creating a hemorrhage-prone cerebral microvasculature. However, also among non-CSVD ICH patients, 40% had hypertension, 50% had at least one vascular risk factor, 50% had advanced global CSVD (score ≥ 2), and even 60% presented with CMB, suggesting an accompanying CSVD pathology in a substantial portion of this subgroup.

Hypertensive arteriopathy caused half of all cases, particularly non-lobar ICH, and was associated with severe vascular risk profiles and advanced hypertensive microvascular pathology on MRI, which is in line with recent studies (2, 15). Chronic hypertension is the most common risk factor of ICH (16, 17) and is responsible for structural and functional maladaptation of cerebral blood vessels, comprising arterial stiffening, hypermuscularization of arteriolar transitional segments, focal smooth muscle cell loss, and impaired myogenic autoregulation. As a result, high-pressure waves are transmitted to vulnerable downstream vessels, promoting the rupture of the vascular wall (18, 19). The high frequency of hypertensive microvascular pathologies on MRI in this subgroup represents a valid predictive potential for individuals at an increased risk for ICH and improves primary prevention, as only half of German or United States hypertensive patients achieve adequate blood pressure control (18, 20). Prospective studies recognized hypertensive microvascular pathologies on MRI as prognostic markers for future ICH and reported a 6-fold increased risk in individuals with deep CMBs (4,500 participants; 5-year follow-up) and a 12-fold increased risk in individuals with severe basal ganglia PVS (1,650 participants; 9-year follow-up), independent of demographics and vascular and genetic risks (21, 22).

Cerebral amyloid angiopathy caused a quarter of ICH cases and half of lobar ICH. Severe CAA, i.e., vascular accumulation of β-amyloid (Aβ), similarly induces morphological changes of the vascular wall, including loss of smooth muscle cells, wall thickening, and lumen restriction, making the abnormally fragile vessels prone to rupture by acute trigger factors like increases in blood pressure or minor trauma (23). As the topographical distribution of CAA mainly affects cortical and leptomeningeal small arteries in lobar brain regions and usually spares deep brain regions and the cerebellum, it is associated with strictly lobar ICH (23, 24). These patients displayed frequent CAA-specific downstream microvascular pathologies on MRI, which were almost absent within the rest of the cohort. In particular, 58% were also diagnosed with hypertension and 43% displayed hypertensive microvascular pathologies on MRI with a HA-CSVD score ≥ 2 (2, 3, 25). Although the arteriolosclerotic/hypertensive brain microvasculature is associated with impaired perivascular clearance of Aβ (18, 26), conventionally, hypertension is seen as an age-related comorbidity in CAA, as human neuropathological studies found no association between hypertension and CAA development. However, individuals with CAA and lobar ICH had more often hypertension than those without ICH, overall suggesting that hypertension in combination with CAA may contribute to CAA-related ICH (23). Evidence from clinical studies supports the causal role of hypertension, as lowering of blood pressure reduced the risk of future CAA-related ICH by approximately 77%, and conversely, inadequate blood pressure control in ICH patients was associated with a 4-fold increased risk for recurrent ICH (27, 28).

Non-CSVD-related ICH etiologies including trauma, vascular malformations, or brain tumors represented the remaining quarter and thus a substantial portion of ICH patients, but are frequently excluded from ICH studies due to their pathophysiological heterogeneity and are therefore insufficiently investigated (14, 29–31). In community-based studies, the prevalence of CMB differs across study populations, ranging from 7 to 18% in adults aged <75 years (32). Here, we report a CMB prevalence of 60% in non-CSVD ICH, mainly consisting of HA-associated deep or mixed CMB (55%). One could speculate that preexisting microvascular hemorrhagic lesions indicate a hemorrhage-prone microcirculation in patients with trauma, vascular malformations, or brain tumors that might aid in stratifying patients who are at increased bleeding risk.

Although the main pathology is the current focus for each ICH etiology, one might consider the possibility of a holistic model, in which a predisposing factor initiates hemorrhagic-prone vascular wall remodeling, i.e., CAA, vascular malformations, brain tumors, or vascular risk factor-driven arteriolosclerosis, to which additional factors can further increase rupture risk, i.e., trauma, antithrombotic medication, or vascular risk factor-driven arteriolosclerosis. This concept is supported by studies, reporting increased hemorrhagic risk under anticoagulation in patients with preexisting vascular risk factors and MRI markers of HA and CAA (33–36). In this context, CSVD severity markers and, particularly, hemorrhagic microvascular lesions on MRI represent a valid readout of systemic microvascular health for non-CSVD-related ICH etiologies as well. For aneurysms and arteriovenous malformations, hypertension and CMB have already been identified as risk factors for rupture of vascular malformations and subsequent hemorrhage (37–42). As more and more unruptured vascular malformations are detected on routine MRI, there is an emerging need for personalized rupture-risk assessment to balance the risk of surgical interventions and conservative waiting. Prospective studies of patients with unruptured vascular malformations may evaluate CSVD burden on MRI to estimate its predictive value for future bleeding risk. Traumatic brain injury leads to ICH in 20% of cases, and preclinical as well as clinical traumatic brain injury studies already suggested that microvascular disease contributes to bleeding risk, while pathophysiological mechanisms remain poorly understood and prognostic markers need to be explored (43, 44). Future retrospective studies that compare CSVD burden between trauma patients with and without ICH could indirectly point to the involvement of CSVD.

Recent studies also highlighted associations between preexisting global CSVD burden, particularly the presence of CMB, and post-ICH disability, dementia as well as depression onset (14, 45). However, these data were entirely derived from CSVD-related ICH, and patients with non-CSVD-related ICH were excluded. There might be a similar prognostic potential of CSVD in the latter group, which needs further exploration. Currently, there is one ongoing prospective study, investigating the effect of preexisting CSVD severity on 12 months of post-traumatic brain injury outcome (46).

### 4.1 Limitations

Our study has limitations, including a descriptive group comparison, due to its retrospective nature, lack of systematic follow-up to obtain outcomes, and the fact that only a subset of patients underwent MRI. The exclusion of aged and severely affected patients who were not suitable for MRI in the clinical setting may have even led to an underestimation of CSVD prevalence. CT scans are still more cost- and time-efficient and can be performed in even severely affected ICH patients. However, the increasing diagnostic and prognostic value of CSVD MRI markers may change their usage in the near future.

In alignment with other retrospective ICH studies, we relied on previous hypertension diagnoses and antihypertensive medication records, which may have underestimated hypertension prevalence (31, 47). Single-hospital blood pressure measures were not considered, as multiple readings on multiple occasions in an ambulatory or at-home setting are recommended for the final diagnosis (7). The final limitation is the relatively uncertain categorization of the underlying arteriopathy in the absence of pathologic data, despite our best efforts to have well-defined groups, including CAA-related purely lobar bleeds vs. purely deep and mixed bleeds, presumably related to HA. Nevertheless, we were able to demonstrate significant associations of typical imaging markers in CSVD that were consistent with previous studies. These drawbacks exist for all large-scale studies that analyze data from ICH cohorts without pathologic confirmation.

### 4.2 Outlook

In conclusion, our study highlights the role of CSVD in ICH development and identifies frequent microvascular pathologies in non-CSVD-related ICH. Our findings may serve as a groundwork to further study the prognostic value of these risk factors for ICH onset and outcome in yet underexamined ICH etiologies.

## Data availability statement

The raw data supporting the conclusions of this article will be made available by the authors, without undue reservation.

## Ethics statement

The local ethic committee of the medical faculty, Otto-von-Guericke University, Magdeburg (No. 28/16, addendum 07/2018) approved this retrospective study. The studies were conducted in accordance with the local legislation and institutional requirements. The participants provided their written informed consent to participate in this study.

## Author contributions

PA: Writing – original draft, Writing – review & editing. CC: Writing – original draft, Writing – review & editing. ML: Writing – review & editing. J-NK: Writing – review & editing. DB: Writing – review & editing. MPf: Writing – review & editing. JN: Writing – review & editing. MG: Writing – review & editing. MD: Writing – review & editing. MPa: Writing – review & editing. RJ: Writing – review & editing. SM: Writing – review & editing. SV: Writing – review & editing. SH: Writing – original draft, Writing – review & editing. SS: Writing – original draft, Writing – review & editing.

## References

[1] Garg R Biller J. Recent advances in spontaneous intracerebral hemorrhage. F1000Res. (2019) 8, 1–3. doi: 10.12688/f1000research.16357.1, PMID: 30906532 PMC6426087

[2] Pasi M Charidimou A Boulouis G Fotiadis P Morotti A Xiong L. Cerebral small vessel disease in patients with spontaneous cerebellar hemorrhage. J Neurol. (2019) 266:625–30. doi: 10.1007/s00415-018-09177-w, PMID: 30617995 PMC9422345

[3] Tsai H-H Pasi M Tsai L-K Chen YF Lee BC Tang SC. Microangiopathy underlying mixed-location intracerebral hemorrhages/microbleeds: a PiB-PET study. Neurology. (2019) 92:e774–81. doi: 10.1212/WNL.0000000000006953, PMID: 30674594 PMC6396971

[4] Greenberg SM Charidimou A. Diagnosis of cerebral amyloid Angiopathy: evolution of the Boston criteria. Stroke. (2018) 49:491–7. doi: 10.1161/STROKEAHA.117.016990, PMID: 29335334 PMC5892842

[5] Dastur CK Yu W. Current management of spontaneous intracerebral haemorrhage. Stroke Vasc Neurol. (2017) 2:21–9. doi: 10.1136/svn-2016-000047, PMID: 28959487 PMC5435209

[6] Yuksen C Sittichanbuncha Y Patumanond J Muengtaweepongsa S Sawanyawisuth K. Clinical predictive score of intracranial hemorrhage in mild traumatic brain injury. Ther Clin Risk Manag. (2018) 14:213–8. doi: 10.2147/TCRM.S147079, PMID: 29440905 PMC5798541

[7] Whelton PK Carey RM Aronow WS Casey DE Jr Collins KJ Dennison Himmelfarb C. 2017 ACC/AHA/AAPA/ABC/ACPM/AGS/APhA/ASH/ASPC/NMA/PCNA guideline for the prevention, detection, evaluation, and Management of High Blood Pressure in adults: a report of the American College of Cardiology/American Heart Association task force on clinical practice guidelines. Hypertension. (2018) 71:e13–e115. doi: 10.1161/HYP.0000000000000065, PMID: 29133356

[8] van Sloten TT Sedaghat S Carnethon MR Launer LJ Stehouwer CDA. Cerebral microvascular complications of type 2 diabetes: stroke, cognitive dysfunction, and depression. Lancet Diabet Endocrinol. (2020) 8:325–36. doi: 10.1016/S2213-8587(19)30405-X, PMID: 32135131 PMC11044807

[9] Wardlaw JM Smith EE Biessels GJ Cordonnier C Fazekas F Frayne R. Neuroimaging standards for research into small vessel disease and its contribution to ageing and neurodegeneration. Lancet Neurol. (2013) 12:822–38. doi: 10.1016/S1474-4422(13)70124-8, PMID: 23867200 PMC3714437

[10] Duering M Biessels GJ Brodtmann A Chen C Cordonnier C de Leeuw FE. Neuroimaging standards for research into small vessel disease-advances since 2013. Lancet Neurol. (2023) 22:602–18. doi: 10.1016/S1474-4422(23)00131-X, PMID: 37236211

[11] Gregoire SM Chaudhary UJ Brown MM Yousry TA Kallis C Jäger HR. The microbleed anatomical rating scale (MARS): reliability of a tool to map brain microbleeds. Neurology. (2009) 73:1759–66. doi: 10.1212/WNL.0b013e3181c34a7d, PMID: 19933977

[12] Wahlund LO Barkhof F Fazekas F Bronge L Augustin M Sjögren M. A new rating scale for age-related white matter changes applicable to MRI and CT. Stroke. (2001) 32:1318–22. doi: 10.1161/01.str.32.6.1318, PMID: 11387493

[13] Potter GM Marlborough FJ Wardlaw JM. Wide variation in definition, detection, and description of lacunar lesions on imaging. Stroke. (2011) 42:359–66. doi: 10.1161/STROKEAHA.110.594754 21193752

[14] Pasi M Sugita L Xiong L Charidimou A Boulouis G Pongpitakmetha T. Association of cerebral small vessel disease and cognitive decline after intracerebral hemorrhage. Neurology. (2021) 96:e182–92. doi: 10.1212/WNL.0000000000011050, PMID: 33067403 PMC7905779

[15] Meretoja A Strbian D Putaala J Curtze S Haapaniemi E Mustanoja S. SMASH-U: a proposal for etiologic classification of intracerebral hemorrhage. Stroke. (2012) 43:2592–7. doi: 10.1161/STROKEAHA.112.661603, PMID: 22858729

[16] Cordonnier C Demchuk A Ziai W Anderson CS. Intracerebral haemorrhage: current approaches to acute management. Lancet. (2018) 392:1257–68. doi: 10.1016/S0140-6736(18)31878-6 30319113

[17] Zille M Farr TD Keep RF Römer C Xi G Boltze J. Novel targets, treatments, and advanced models for intracerebral haemorrhage. EBioMedicine. (2022) 76:103880. doi: 10.1016/j.ebiom.2022.103880, PMID: 35158309 PMC8850756

[18] Ungvari Z Toth P Tarantini S Prodan CI Sorond F Merkely B. Hypertension-induced cognitive impairment: from pathophysiology to public health. Nat Rev Nephrol. (2021) 17:639–54. doi: 10.1038/s41581-021-00430-6, PMID: 34127835 PMC8202227

[19] Ratelade J Klug NR Lombardi D Angelim MKSC Dabertrand F Domenga-Denier V. Reducing Hypermuscularization of the transitional segment between arterioles and capillaries protects against spontaneous intracerebral hemorrhage. Circulation. (2020) 141:2078–94. doi: 10.1161/CIRCULATIONAHA.119.040963, PMID: 32183562 PMC7311305

[20] Neuhauser HK Adler C Rosario AS Diederichs C Ellert U. Hypertension prevalence, awareness, treatment and control in Germany 1998 and 2008-11. J Hum Hypertens. (2015) 29:247–53. doi: 10.1038/jhh.2014.82, PMID: 25273858

[21] Akoudad S Portegies MLP Koudstaal PJ Hofman A van der Lugt A Ikram MA. Cerebral microbleeds are associated with an increased risk of stroke: the Rotterdam study. Circulation. (2015) 132:509–16. doi: 10.1161/CIRCULATIONAHA.115.016261 26137955

[22] Duperron M-G Tzourio C Schilling S Zhu YC Soumaré A Mazoyer B. High dilated perivascular space burden: a new MRI marker for risk of intracerebral hemorrhage. Neurobiol Aging. (2019) 84:158–65. doi: 10.1016/j.neurobiolaging.2019.08.031 31629114

[23] Charidimou A Gang Q Werring DJ. Sporadic cerebral amyloid angiopathy revisited: recent insights into pathophysiology and clinical spectrum. J Neurol Neurosurg Psychiatry. (2012) 83:124–37. doi: 10.1136/jnnp-2011-301308, PMID: 22056963

[24] Charidimou A Boulouis G Frosch MP Baron JC Pasi M Albucher JF. The Boston criteria version 2.0 for cerebral amyloid angiopathy: a multicentre, retrospective. Neurology. (2022) 21:714–25. doi: 10.1016/S1474-4422(22)00208-3, PMID: 35841910 PMC9389452

[25] Zhang S Wang Z Zheng A Yuan R Shu Y Zhang S. Blood pressure and outcomes in patients with different etiologies of intracerebral hemorrhage: a multicenter cohort study. J Am Heart Assoc. (2020) 9:e016766. doi: 10.1161/JAHA.120.016766, PMID: 32924756 PMC7792400

[26] Jandke S Garz C Schwanke D Sendtner M Heinze HJ Carare RO. The association between hypertensive arteriopathy and cerebral amyloid angiopathy in spontaneously hypertensive stroke-prone rats. Brain Pathol. (2018) 28:844–59. doi: 10.1111/bpa.12629, PMID: 30062722 PMC8028507

[27] Arima H Tzourio C Anderson C Woodward M Bousser MG MacMahon S. Effects of perindopril-based lowering of blood pressure on intracerebral hemorrhage related to amyloid angiopathy: the PROGRESS trial. Stroke. (2010) 41:394–6. doi: 10.1161/STROKEAHA.109.563932, PMID: 20044530

[28] Biffi A Anderson CD Battey TWK Ayres AM Greenberg SM Viswanathan A. Association between blood pressure control and risk of recurrent intracerebral hemorrhage. JAMA. (2015) 314:904–12. doi: 10.1001/jama.2015.10082, PMID: 26325559 PMC4737594

[29] Yakushiji Y Tanaka J Wilson D Charidimou A Noguchi T Kawashima M. Proportion of intracerebral haemorrhage due to cerebral amyloid angiopathy in the east and west: comparison between single hospital centres in Japan and the United Kingdom. J Neurol Sci. (2020) 416:117037. doi: 10.1016/j.jns.2020.117037, PMID: 32711192

[30] Lioutas V-A Beiser AS Aparicio HJ Himali JJ Selim MH Romero JR. Assessment of incidence and risk factors of intracerebral hemorrhage among participants in the Framingham heart study between 1948 and 2016. JAMA Neurol. (2020) 77:1252–60. doi: 10.1001/jamaneurol.2020.1512, PMID: 32511690 PMC7281354

[31] Charidimou A Boulouis G Haley K Auriel E van Etten ES Fotiadis P. White matter hyperintensity patterns in cerebral amyloid angiopathy and hypertensive arteriopathy. Neurology. (2016) 86:505–11. doi: 10.1212/WNL.0000000000002362, PMID: 26747886 PMC4753727

[32] Vernooij MW van der Lugt A Ikram MA Wielopolski PA Niessen WJ Hofman A. Prevalence and risk factors of cerebral microbleeds: the Rotterdam scan study. Neurology. (2008) 70:1208–14. doi: 10.1212/01.wnl.0000307750.41970.d9 18378884

[33] Schreiber S Wilisch-Neumann A Schreiber F Assmann A Scheumann V Perosa V. Invited review: the spectrum of age-related small vessel diseases: potential overlap and interactions of amyloid and nonamyloid vasculopathies. Neuropathol Appl Neurobiol. (2020) 46:219–39. doi: 10.1111/nan.12576, PMID: 31386773

[34] Gattringer T Eppinger S Beitzke M Wuensch G Niederkorn K Deutschmann H. Cortical superficial Siderosis and risk of bleeding after thrombolysis for ischemic stroke. Cerebrovasc Dis. (2015) 40:191–7. doi: 10.1159/000439184, PMID: 26351845

[35] Charidimou A Shoamanesh A. Clinical relevance of microbleeds in acute stroke thrombolysis: comprehensive meta-analysis. Neurology. (2016) 87:1534–41. doi: 10.1212/WNL.0000000000003207, PMID: 27629086

[36] Das AS Gökçal E Regenhardt RW Warren AD Biffi A Goldstein JN. Clinical and neuroimaging risk factors associated with the development of intracerebral hemorrhage while taking direct oral anticoagulants. J Neurol. (2022) 269:6589–96. doi: 10.1007/s00415-022-11333-2, PMID: 35997817 PMC10947801

[37] Greving JP Wermer MJH Brown RD Morita A Juvela S Yonekura M. Development of the PHASES score for prediction of risk of rupture of intracranial aneurysms: a pooled analysis of six prospective cohort studies. Lancet Neurol. (2014) 13:59–66. doi: 10.1016/S1474-4422(13)70263-1, PMID: 24290159

[38] Langer DJ Lasner TM Hurst RW Flamm ES Zager EL King JT Jr. Hypertension, small size, and deep venous drainage are associated with risk of hemorrhagic presentation of cerebral arteriovenous malformations. Neurosurgery. (1998) 42:481–6. doi: 10.1097/00006123-199803000-00008, PMID: 9526981

[39] Tong X Wu J Lin F Cao Y Zhao Y Ning B. Brain arteriovenous malformations in elderly patients: clinical features and treatment outcome. Acta Neurochir. (2015) 157:1645–54. doi: 10.1007/s00701-015-2521-6 26276468

[40] Zhang X Yao Z-Q Karuna T Duan CZ Wang XM Li XF. Cerebral microbleeds could be independently associated with intracranial aneurysm rupture: a cross-sectional population-based study. World Neurosurg. (2018) 115:e218–25. doi: 10.1016/j.wneu.2018.04.018, PMID: 29654957

[41] Guo Y Saunders T Su H Kim H Akkoc D Saloner DA. Silent intralesional microhemorrhage as a risk factor for brain arteriovenous malformation rupture. Stroke. (2012) 43:1240–6. doi: 10.1161/STROKEAHA.111.647263, PMID: 22308253 PMC3335931

[42] Stapf C Mast H Sciacca RR Choi JH Khaw AV Connolly ES. Predictors of hemorrhage in patients with untreated brain arteriovenous malformation. Neurology. (2006) 66:1350–5. doi: 10.1212/01.wnl.0000210524.68507.87, PMID: 16682666

[43] Perel P Roberts I Bouamra O Woodford M Mooney J Lecky F. Intracranial bleeding in patients with traumatic brain injury: a prognostic study. BMC Emerg Med. (2009) 9:15. doi: 10.1186/1471-227X-9-15, PMID: 19650902 PMC2735732

[44] Kenney K Amyot F Haber M Pronger A Bogoslovsky T Moore C. Cerebral vascular injury in traumatic brain injury. Exp Neurol. (2016) 275:353–66. doi: 10.1016/j.expneurol.2015.05.019 26048614

[45] Castello JP Pasi M Kubiszewski P Abramson JR Charidimou A Kourkoulis C. Cerebral small vessel disease and depression among intracerebral hemorrhage survivors. Stroke. (2022) 53:523–31. doi: 10.1161/STROKEAHA.121.035488, PMID: 34587793 PMC8792169

[46] Salomon G Barrett K. Impact of cerebral small vessel disease on neurological outcomes following traumatic brain injury (P 15-6. 003). Neurology. (2022) 98:3401. doi: 10.1212/WNL.98.18_supplement.3401

[47] Charidimou A Boulouis G Pasi M Auriel E van Etten ES Haley K. MRI-visible perivascular spaces in cerebral amyloid angiopathy and hypertensive arteriopathy. Neurology. (2017) 88:1157–64. doi: 10.1212/WNL.0000000000003746, PMID: 28228568 PMC5373782

