# Supplementary material for: Risk factors for intracerebral hemorrhage in small-vessel disease and non-small-vessel disease etiologies—an observational proof-of-concept study
Source: Front Neurol. 2024 Mar 7;15:1322442. doi: 10.3389/fneur.2024.1322442 (PMC10954881; doi:10.3389/fneur.2024.1322442)
Supplement: Supplementary file 1 [file Data_Sheet_1.pdf]

Supplementary Material

Supplementary Table 1. Demographics, clinical data and ICH location in patients with/without vascular imaging. Vascular imaging comprised computer tomography angiography, magnetic resonance angiography and digital subtraction angiography. Data are presented as median (IQR) or number (%). Percentage is given in relation to the specific subgroup. Group comparison was conducted applying Mann Whitney U-test for continuous / ordinal variables and $\chi 2$-test for categorial variables. A p-value < 0.05 was considered significant and highlighted in bold. Patients who underwent vascular imaging were younger and less often under a combination of anticoagulant and antiplatelet medication compared to those without vascular imaging.

[a] Percentage is given in relation to number of dyslipidemia patients. CAA, cerebral amyloid angiopathy; HA, hypertensive arteriopathy; IHC, intracerebral hemorrhage; IQR, interquartile range; VM, vascular malformations.

**Demographics**	**Vascular imaging** *n = 729*	**No vascular imaging** *n = 193*	p-value
Age, median (IQR)	70 (59-77)	72 (61-79)	**0.04**
Male sex, n (%)	410 (56%)	112 (58%)	0.66
**Cardiovascular risk**	*n = 689*	*n = 173*	
Cardiovascular risk sum score, median (IQR)	0.33 (0.00-0.67)	0.33 (0.00-0.67)	0.67
Three vascular risk factors, n (%)	57 (8%)	14 (8%)	0.75
Hypertension, n (%)	361 (53%)	89 (51%)	0.81
Intake of > 1 anti-hypertensive drug, n (%)	241 (35%)	59 (34%)	0.97
Dyslipidemia, n (%)	203 (30%)	50 (29%)	0.88
Intake of statins, n (%[a])	115 (57%)	35 (70%)	0.09
Type 2 diabetes, n (%)	159 (23%)	38 (22%)	0.75
**Antithrombotics**	*n = 689*	*n = 173*	
Antithrombotics, n (%)	203 (30%)	55 (32%)	0.59
Anticoagulants, n (%)	115 (16%)	34 (18%)	0.54
Antiplatelets, n (%)	82 (11%)	15 (8%)	0.16
Antiplatelets and anticoagulants, n (%)	6 (1%)	6 (3%)	**0.01**
**Location**	*n = 729*	*n = 193*	
Lobar	328 (45%)	86 (45%)	0.96
Deep	260 (36%)	61 (32%)	0.26
Cerebellar	86 (12%)	31 (16%)	0.11
Ventricular	55 (8%)	15 (8%)	0.92
**Etiology**	*n = 729*	*n = 193*	
HA (%)	347 (48%)	95 (49%)	0.79
CAA (%)	189 (26%)	39 (21%)	0.14
Non-CSVD	193 (26%)	59 (31%)	0.26

Supplementary Table 2. Demographics, clinical data, ICH location and etiology in patients with/without MRI. Data are presented as median (interquartile range) or number (%). Percentage is given in relation to the specific subgroup. Group comparison was conducted applying Mann Whitney U-test for continuous / ordinal variables and $\chi 2$-test for categorial variables. A p-value < 0.05 was considered significant and highlighted in bold. Patients who underwent MRI compared to those without MRI had more often non-CSVD ICH, less often HA-related ICH and ICH location was more often lobar and less often in deep brain regions. Patients, who received MRI, were younger and less often under anticoagulant and statin medication. There were no differences in sex and vascular risk

[a] Percentage is given in relation to number of dyslipidemia patients. CAA, cerebral amyloid angiopathy; HA, hypertensive arteriopathy; IHC, intracerebral hemorrhage; MRI, magnetic resonance imaging; VM, vascular malformations.

	**MRI**	**No MRI**	p-value
**Demographics**	*n = 223*	*n = 703*	
Age, median (IQR)	68 (54-75)	71 (61-78)	**< 0.0001**
Male sex, n (%)	129 (58%)	395 (56%)	0.66
**Cardiovascular risk**	*n = 208*	*n = 653*	
Cardiovascular risk sum score, median (IQR)	0.33 (0.00-0.67)	0.33 (0.00-0.67)	0.71
Three vascular risk factors, n (%)	20 (10%)	51 (8%)	0.84
Hypertension, n (%)	108 (51%)	343 (53%)	0.69
Intake of > 1 anti-hypertensive drug, n (%)	62 (30%)	238 (36%)	0.07
Dyslipidemia, n (%)	73 (34%)	182 (28%)	0.07
Intake of statins, n (%[a])	35 (48%)	115 (63%)	**0.04**
Type 2 diabetes, n (%)	48 (23%)	152 (23%)	0.85
**Antithrombotics**	*n = 208*	*n = 653*	
Antithrombotics, n (%)	59 (27%)	200 (29%)	0.42
Anticoagulants, n (%)	24 (11%)	125 (18%)	**0.01**
Antiplatelets, n (%)	31 (14%)	67 (10%)	0.06
Antiplatelets and anticoagulants, n (%)	4 (2%)	8 (1%)	0.45
**Location**	*n = 223*	*n = 703*	
Lobar	120 (54%)	298 (42%)	**0.003**
Deep	60 (27%)	259 (37%)	**0.007**
Cerebellar	30 (14%)	88 (13%)	0.72
Ventricular	13 (6%)	58 (8%)	0.24
**Etiology**	*n = 223*	*n = 703*	
HA (%)	77 (36%)	367 (52%)	**< 0.0001**
CAA (%)	58 (26%)	172 (25%)	0.64
Non-CSVD	88 (40%)	164 (23%)	**< 0.0001**

Supplementary Table 3. Demographics, clinical data and etiology in different ICH location subgroups. Data are presented as median (interquartile range) or number (%). Percentage is given in relation to the specific subgroup. Group comparisons between locations of ICH were conducted applying Kruskal-Wallis-test for continuous / ordinal variables and $\chi 2$-test for categorial variables. P-values from pairwise post-hoc tests were Bonferroni adjusted for multiple comparisons. For categorical variables the post hoc test was used to identify which particular subgroups were significantly different from the entire cohort. A (adjusted) p-value ≤ 0.05 was considered significant and highlighted in bold.

[a]Percentage is given in relation to number of dyslipidemia patients. CAA, cerebral amyloid angiopathy; CSVD, cerebral small vessel disease; HA, hypertensive arteriopathy; ICH, intracerebral hemorrhage.

	**Total**	**Lobar**	**Deep**	**Cerebellar**	**Ventricular**	*Group analysis*	*Subgroup comparisons*
**Demographics**	*n = 922*	*n = 403*	*n = 332*	*n = 117*	*n = 70*	*p-value*	*p-value*
Age	71 (60-78)	71 (57-79)	70 (60-77)	71 (64-77)	69 (54-77)	p = 0.36	
Male sex	522 (57%)	230 (56%)	197 (61%)	62 (53%)	33 (47%)	p = 0.10	
**Cardiovascular risk**	*n = 862*	*n = 382*	*n = 304*	*n = 108*	*n = 68*		
Vascular risk sum score	0.33 (0.00-0.67)	0.33 (0.00-0.67)	0.33 (0.00-0.67)	0.33 (0.00-0.67)	0.33 (0.00-0.67)	**p = 0.046**	***Lobar vs. Deep: adj. p = 0.047***
Three vascular risk factors	71 (8%)	20 (5%)	37 (12%)	9 (8%)	5 (7%)	**p = 0.01**	***Deep: adj. p = 0.01***
Hypertension	450 (52%)	189 (50%)	163 (54 %)	64 (59%)	34 (50%)	p = 0.31	
≥ 2 anti-hypertensive drugs	300 (35%)	109 (29%)	117 (38%)	48 (44%)	26 (38%)	**p = 0.005**	***Lobar: adj. p = 0.005***
Dyslipidemia	253 (29%)	105 (28%)	105 (35%)	28 (26%)	15 (22%)	p = 0.08	
Intake of statins[a]	150 (59%)	74 (71%)	46 (44%)	17 (61%)	13 (87%)	**p = 0.0001**	***Lobar: adj. p = 0.02***<br>***Deep: adj. p = 0.0002***
Type 2 diabetes	197 (23%)	65 (17%)	82 (27%)	29 (27%)	21 (31%)	**p = 0.003**	***Lobar: adj. p = 0.003***
**Antithrombotics**	*n = 862*	*n = 382*	*n = 304*	*n = 108*	*n = 68*		
Antithrombotics	258 (30%)	113 (30%)	95 (31%)	26 (24%)	24 (35%)	p = 0.40	
Anticoagulants	149 (17%)	60 (16%)	48 (16%)	21 (21%)	20 (29%)	**p = 0.02**	***Ventricular: adj. p = 0.03***
Antiplatelets	97 (11%)	47 (12%)	42 (14%)	4 (4%)	4 (6%)	**p = 0.02**	
Anticoagulants and Antiplatelets	12 (1%)	6 (2%)	5 (2%)	1 (1%)	0 (0%)	p = 0.72	
**Etiology**	*n = 922*	*n = 403*	*n = 332*	*n = 117*	*n = 70*		
HA	453 (49%)	0 (0%)	309 (93%)	94 (80%)	50 (71%)	**p < 0.0001**	***All subgroups: adj. p < 0.001***
CAA	228 (25%)	217 (54%)	0 (0%)	0 (0%)	0 (0%)	**p < 0.0001**	***All subgroups: adj. p < 0.0001***
Non-CSVD	252 (27%)	186 (46%)	23 (7 %)	23 (20%)	20 (29%)	**p < 0.0001**	***Lobar: adj. p < 0.0001***<br>***Deep: adj. p < 0.0001***

Supplementary Table 4. Quantification of CSVD severity according to STRIVE in ICH due to different non-CSVD etiologies. Data are presented as median (interquartile range) or number (%). Percentage is given in relation to the specific subgroup. Group comparisons between vascular malformation-, traumatic brain injury- and brain tumor-related ICH were conducted applying Kruskal-Wallis-test for continuous / ordinal variables and χ2-test for categorial variables. A p-value ≤ 0.05 was considered significant and highlighted in bold.

CMB, cerebral microbleeds; CSVD, cerebral small vessel disease; PVS, perivascular spaces; ICH, intracerebral hemorrhage; WMH, white matter hyperintensities.

	**Vascular malformation** *n = 37*	**Traumatic brain injury** *n = 13*	**Brain tumor** *n = 27*	*Group analysis* *p-value*
Global CSVD score	2 (0-2)	1 (0-3)	2 (0-2)	*p = 0.98*
**Cerebral microbleeds**				
Number of CMB	4 (0-9)	0 (0-15)	5 (0-13)	*p = 0.89*
Strictly lobar CMB	3 (8%)	1 (8%)	0 (0%)	*p = 0.32*
Strictly deep CMB	7 (19%)	2 (15%)	4 (15%)	*p = 0.90*
Mixed CMB	13 (35%)	4 (31%)	12 (44%)	*p = 0.64*
**White matter hyperintensities**				
Periventricular WMH score	1 (0-1)	1 (0-2)	1 (0-2)	*p = 0.49*
Deep WMH score	0 (0-1)	0 (0-1)	0 (0-1)	*p = 0.77*
**Perivascular spaces**				
Basal ganglia PVS score	1 (1-1)	1 (1-2)	1 (1-1)	*p = 0.12*
Severe basal ganglia PVS (>20)	0 (0%)	0 (0%)	0 (0%)	*p = 1.00*
Centrum semiovale PVS score	1 (1-2)	1 (1-2)	1 (1-2)	*p = 0.94*
Severe centrum semiovale PVS (>20)	3 (8%)	0 (0%)	1 (4%)	*p = 0.48*
**Lacunes**				
Lacune presence	3 (8%)	2 (15%)	1 (4%)	*p = 0.43*
**Cortical superficial siderosis**				
Presence of cortical superficial siderosis	1 (3%)	0 (0%)	0 (0%)	*p = 0.98*

Supplementary Table 5. Multivariable linear regression analysis identifies predictors of CSVD severity in patients with ICH. Multivariable linear regression analyses were performed to test for independent associations with different CSVD markers on MRI, including ICH etiology, demographics and cardiovascular risk. Stepwise forward variable selection ($p < 0.05$) was subsequently used to generate a minimal adjusted model. Data represent standardized effects estimates (β) and the corresponding p-value. Associations achieving statistical significance ($p < 0.05$) are highlighted in bold.

BG, basal ganglia; CAA, cerebral amyloid angiopathy; CMB, cerebral microbleed; CSO, centrum semiovale; CSVD, cerebral small vessel disease; cSS, cortical superficial siderosis; CSVD, cerebral small vessel disease; EPVS, enlarged perivascular space; HA, hypertensive arteriopathy; ICH, intracerebral hemorrhage; WMH, white matter hyperintensities.

	**Lacune number**		**Deep WMH**		**BG EPVS score**		**CMB number**	
**Vascular risk profile**	*β*	*p-value*	*β*	*p-value*	*β*	*p-value*	*β*	*p-value*
Hypertension	0.22	**0.004**	0.05	0.45	0.09	0.27	0.08	0.31
Intake of > 1 antihypertensive drugs	-0.06	0.50	0.05	0.49	0.19	**0.003**	0.09	0.20
Dyslipidemia	0.00	0.96	-0.10	0.13	-0.02	0.77	-0.05	0.59
Intake of statins	0.04	0.59	-0.05	0.41	-0.03	0.68	0.18	**0.013**
Type 2 diabetes	0.16	**0.03**	0.04	0.57	0.01	0.89	0.16	**0.025**
**Covariates**								
HA-ICH	0.07	0.36	0.28	**< 0.0001**	0.34	**< 0.0001**	0.12	0.13
CAA-ICH	0.01	0.91	0.35	**< 0.0001**	0.27	**< 0.0001**	0.32	**< 0.0001**
Age	-0.04	0.63	0.37	**< 0.0001**	0.30	**< 0.0001**	0.12	0.13
Male sex	0.05	0.47	-0.02	0.76	-0.04	0.50	0.09	0.18

	**cSS score**		**Periventricular WMH**		**CSO EPVS score**	
**Vascular risk profile**	β	p-value	β	p-value	β	p-value
Hypertension	0.00	0.99	0.10	0.15	-0.02	0.84
Intake of > 1 antihypertensive drugs	-0.14	**0.046**	0.02	0.75	-0.08	0.25
Dyslipidemia	-0.10	0.18	-0.05	0.45	-0.01	0.88
Intake of statins	-0.03	0.67	-0.05	0.46	-0.05	0.47
Type 2 diabetes	-0.10	0.08	0.06	0.38	-0.03	0.68
**Covariates**						
HA-ICH	0.00	0.97	0.33	**< 0.0001**	0.21	**0.006**
CAA-ICH	0.43	**< 0.0001**	0.35	**< 0.0001**	0.46	**< 0.0001**
Age	0.09	0.22	0.37	**< 0.0001**	0.17	**0.017**
Male sex	-0.01	0.91	-0.03	0.65	-0.13	0.056
